# The key role of 3D printing and the new medical sterilizable threads in the development of the translaryngeal Tracheostomy Needle Introducer

**DOI:** 10.1186/s41205-021-00104-w

**Published:** 2021-05-12

**Authors:** Alessandro Terrani, Enrico Bassi, Alberto Ornaghi, Giacomo Bellani, Giuseppe Foti

**Affiliations:** 1 Department of Emergency and Intensive Care, ASST-Brianza, Desio Hospital, Via Mazzini 1, Desio, Italy; 2 Designer, Opendot Lab, Via Tertulliano 70, Milan, Italy; 3 Department of Emergency and Intensive Care, ASST-Monza, Monza, Italy; 4 School of Medicine and Surgery, University of Milan-Bicocca, Via Cadore 48, Monza, Italy

**Keywords:** 3D printing, Tracheostomy, Percutaneous, Translaryngeal tracheostomy, Needle introducer, Additive manufacturing technologies, Digital fabrication, Material extrusion

## Abstract

**Background:**

Percutaneous tracheostomy is frequently performed in intensive care units in patients who require prolonged mechanical ventilation. The first crucial step for the physician in these procedures is the precise needle insertion into the trachea. The primary aim of this technical note was to test the new filament and share our experiences in the implementation of the new device. The secondary aim was to show how a physician with basic training in computer-aided design and three-dimensional (3D) printing could independently create useful devices for clinical practice.

**Methods:**

To simplify this referred clinical procedure and increase its safety, 3D printing and a new medical filament were used to develop a new translaryngeal Tracheostomy Needle Introducer (tTNI) for use in conjunction with the Fantoni’s method of percutaneous tracheostomy. The tTNI is composed of three parts: a support to fit on the rigid endotracheal tube of the Fantoni kit, an external particular shaped arm, and an introducer for the needle. The latest version of the device used a new filament based on a polyester matrix certified for skin contact that was sterilizable in a standard autoclave. Post-printing, minor technical interventions were required to correct small material deformities.

**Conclusions:**

Our experiences with the thread and the technical features of the material were reported herein in conjunction with some suggestions on how to solve the most frequently encountered problems. The 3D printing technique allows physicians to directly manage the prototyping process of new medical devices, making this process completely independent. The speed of the prototyping process and the testing of each piece allow faster creation of a prototype than with traditional industrial methods. Finally, the new biomedical filaments offer endless possibilities of creation and modelling.

**Supplementary Information:**

The online version contains supplementary material available at 10.1186/s41205-021-00104-w.

## Background

In all of the currently used percutaneous tracheostomy techniques [1–3], the first crucial step is the precise needle insertion into the trachea. Specifically, different devices have been developed for precise needle insertion in the trachea [4–7]. However, to our knowledge, none of the devices fulfils all the essential safety standards. These include a continuous endoscopic view of the trachea during the procedure, avoidance of the use of a metal stylet in the tracheal lumen, and prevention of a device from extending beyond the distal end of the endotracheal tube.

Based on the technical need to solve this issue and aiming to simplify the procedure and increase its safety, three-dimensional (3D) printing was used to develop the translaryngeal Tracheostomy Needle Introducer (tTNI, Italian patent number 102017000035827, see supplementary materials) for use in conjunction with Fantoni’s kit of percutaneous tracheostomy [8, 9].

To implement a device that allows the safe introduction of the needle into the trachea, the team was inspired by the Stryker Gamma3 long nail system (Stryker Corporation, Kalamazoo, MI, USA) for femoral fractures [10]. An external mechanical arm (targeting arm) was used to precisely guide the screws which blocked the endomedullary nail to distances of the order of millimetres from the correct position. Based on the same principle, an external arm was developed to guide tracheal needle insertion based on the rigidity and known measurements of the tracheoscope used for Fantoni’s procedure (Covidien-DAR, Medtronic, Minneapolis, MN, USA).

To conduct clinical trials with tTNI, it was necessary to identify a filament that was biocompatible and sterilizable. After extensive research, a new thread, referred to as Verum T, the 3D printer filament based on a polyester matrix and certified for skin contact, was found to be compatible for use to address our set purposes [11]. Therefore, the team was able to construct the final version of the tTNI entirely composed of new thread to be ready for clinical use.

The main aim of the study was to test the new filament and share our experience in creating the final version of the tTNI.

The secondary aim was to show how a physician with a basic training in computer-aided design could independently and easily create devices useful for his clinical needs with a 3D printer and appropriate threads.

## Methods

The tTNI is composed of three parts: a support to fit on the rigid endotracheal tube of the Fantoni kit, an external shaped arm, and an introducer for the 18 Gauge angiographic needle with a length of 7 cm. Versions 1.0 and 2.0 were designed using the software Rhinoceros (Robert McNeel and Associates, Seattle, WA, USA) and made using digital fabrication technologies (3D printer Ultimaker 2+ and laser cutter GCC) in the OpenDot (Milano, Italy) laboratory.

**Fig. 1:**
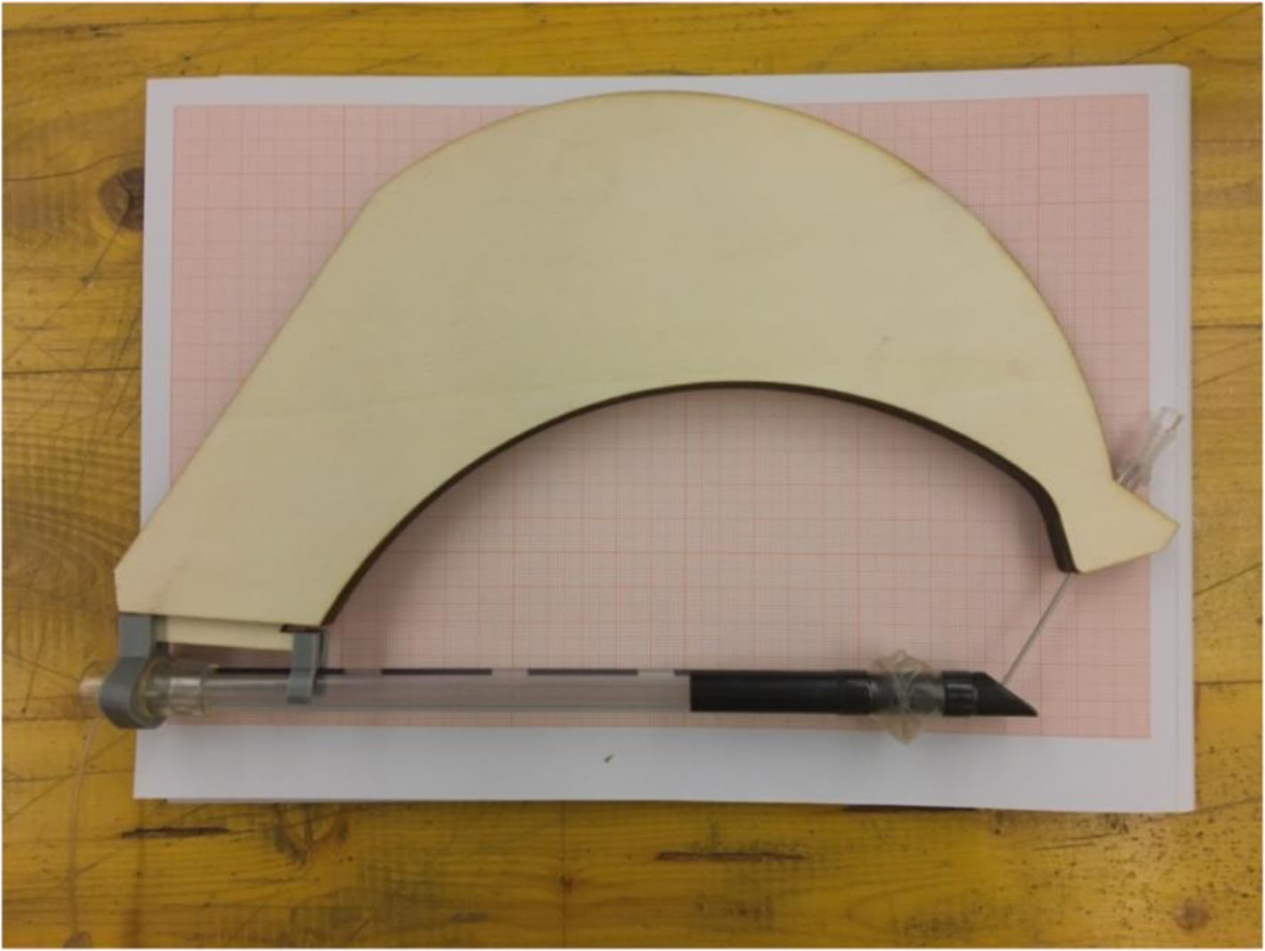
Version 1.0 of the tTNI made in plywood and PLA

**Fig. 2:**
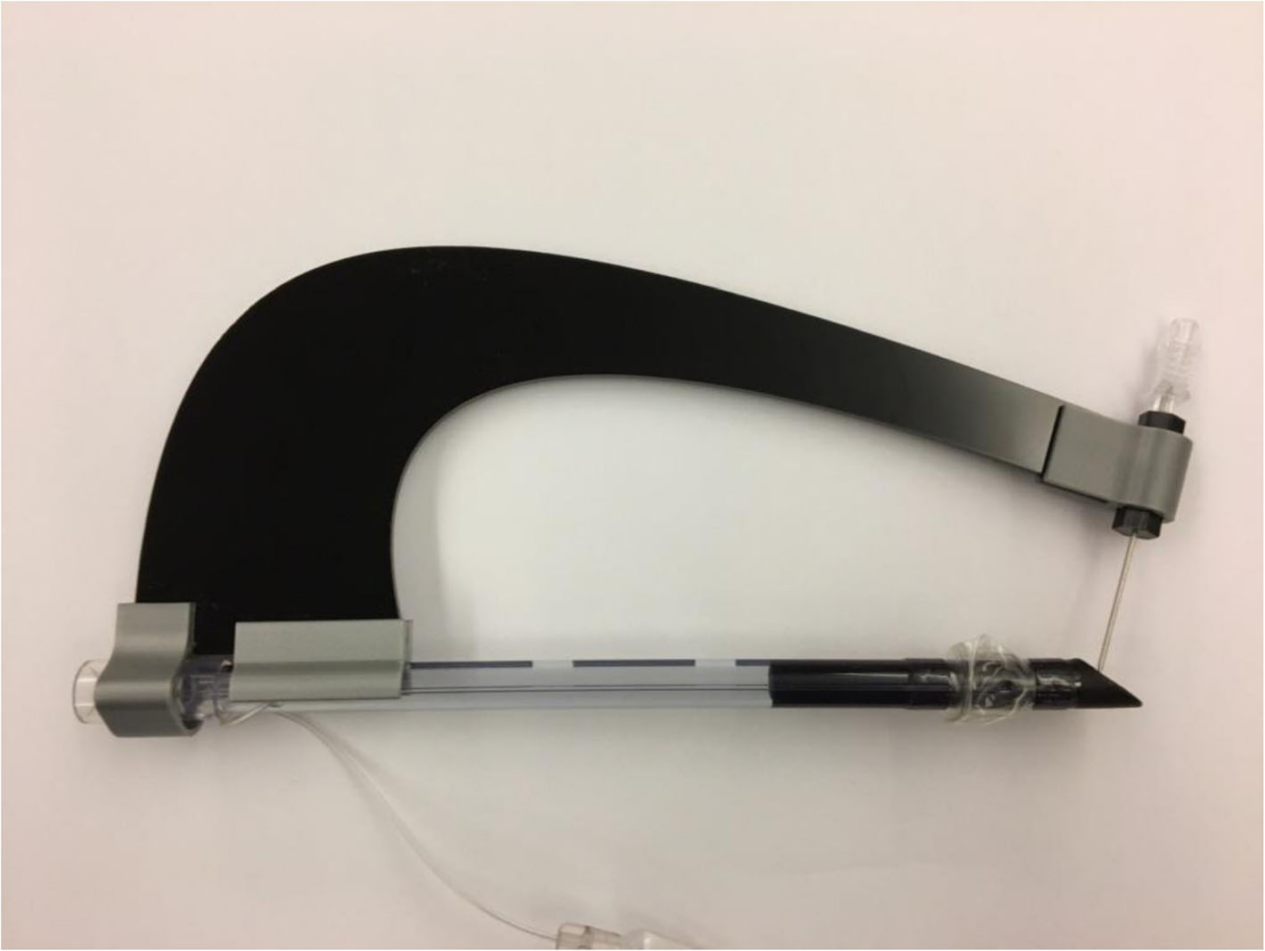
Version 2.0 made of plexiglass and PLA

Version 1.0 of the tTNI was made with two PLA (polylactic acid) supports and a plywood arm (Fig. 1), was tested and improved after extensive trials on a mannequin for advanced life support and tracheostomy training. This led to the second version (Version 2.0) made of two PLA supports and an introducer with a plexiglass arm (Fig. 2).

**Fig. 3:**
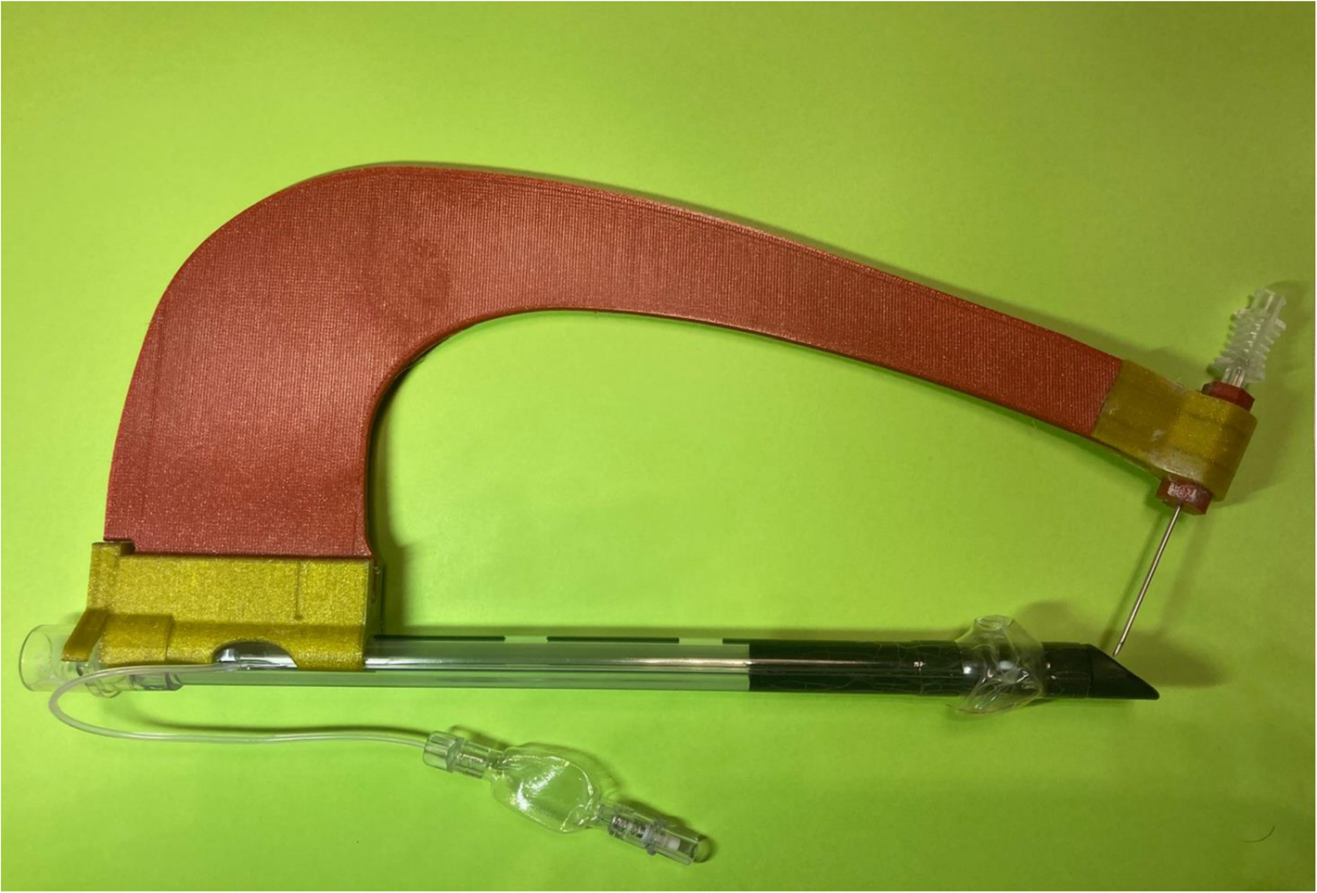
Version 2.1 and technical design of the tTNI


To improve the fitting of the support on the rigid endotracheal tube of Fantoni’s kit, and the fitting of the arm on the support, version 2.1 was developed with a single and longer PLA support (Fig. 3). The detailed photographs of all parts of the device, and the video on the assembly of each piece are listed in the supplementary materials.

**Additional file 3.** Supplemental_tTNI_Video.

The tTNI version 2.1 has been tested in a Canadian cadaver lab with very good results regarding the safety and precision of the tracheal puncture performed. The use of the tTNI has minimised the number of attempts for correct positioning of the needle in the trachea in a shorter time period for both experienced and untrained physicians [12].

To proceed to clinical trials, it was necessary to construct the device with a filament that is biocompatible and sterilizable. After extensive research, the Verum T® 3D printer filament® (Treed Filaments, Seregno, Italy), which was based on a polyester matrix and certified for skin contact (according to the UNI, EN, and ISO10993-5 standards) and which could be sterilized in a standard autoclave, was found to be compatible for use to address our set purposes.

The manufacturer has granted the necessary certification for the product but for the conduct of the individual tests performed (please see the supplementary materials section). The company possesses all the documents related to the safety certification to those who directly request it after the signature of a nondisclosure agreement.

**Fig. 4:**
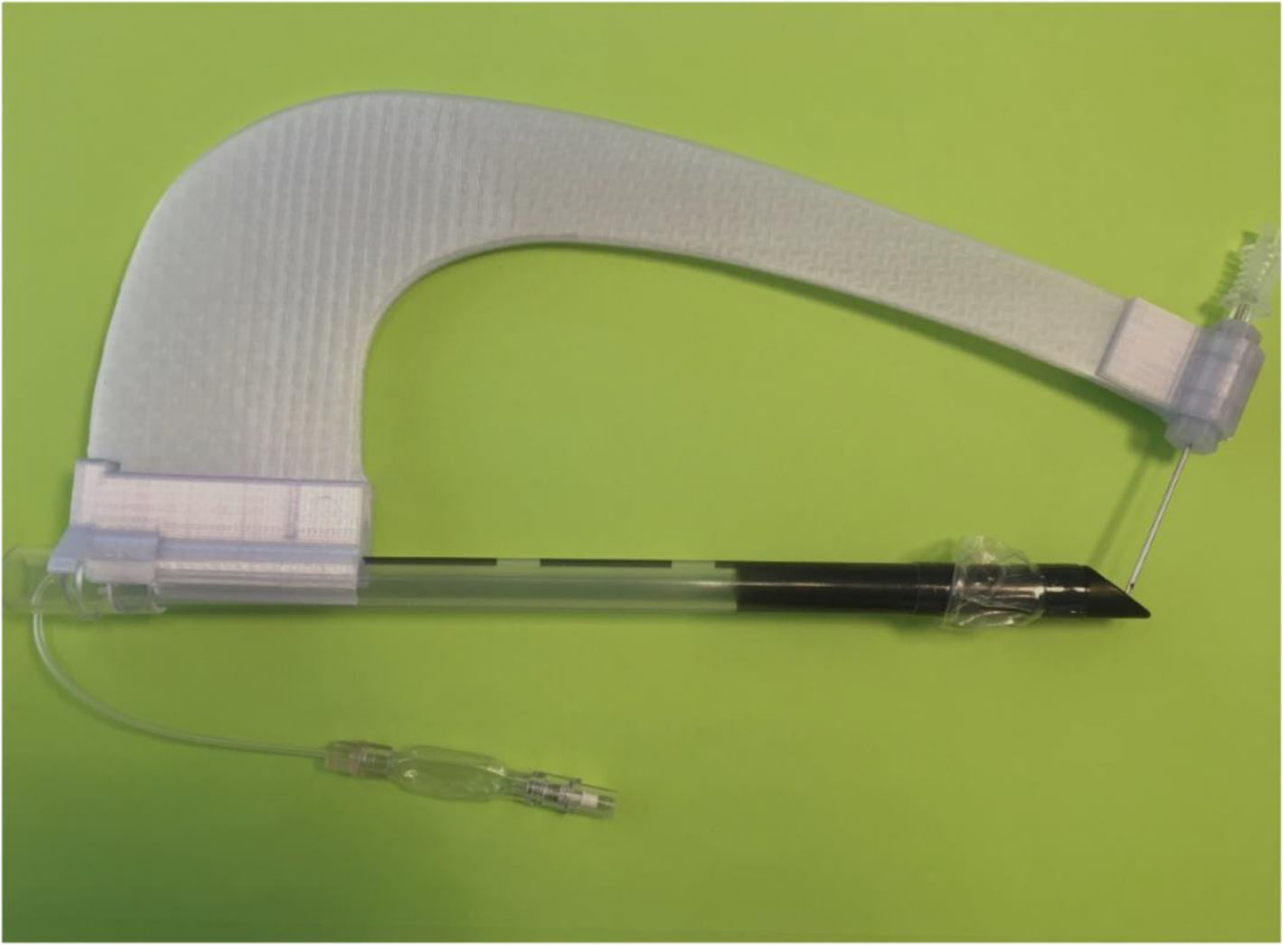
tTNI v3.0 constructed entirely of Verum T

Therefore, it has been possible to implement version 3.0 (Fig. 4) of the tTNI, which was entirely made of sterilizable and biocompatible material so as to be ready for clinical use.

**Fig. 5:**
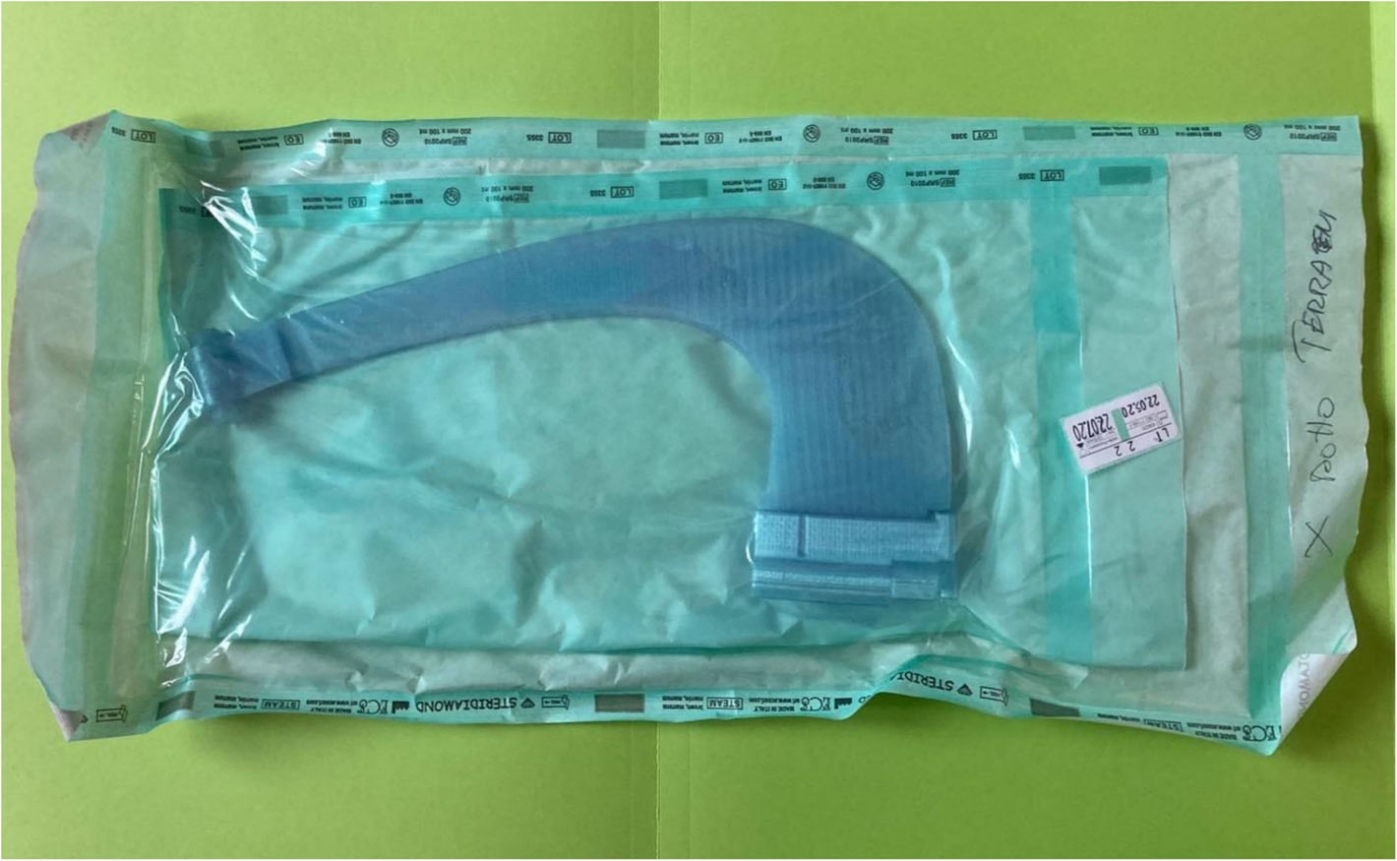
Photograph of tTNI v3.0 after the sterilization process

The device was then sterilized in a STERRAD-NX autoclave at 50 °C for 28 min (see Fig. 5). At the end of the sterilization process, the device worked normally, and no alterations or deformations were identified. After sterilization, we re-tested the devices several times on the same mannequins and found no changes in the puncture sites. No other mechanical tests were performed.

Version 3.0 was designed using Autodesk Fusion 360 software (Autodesk, Mill Valley, CA, USA) and was constructed using a Prusa I3 MK3S printer (Prusa Research, Prague, Czechia) [13] by Dr. Alessandro Terrani (Intensivist physician with an interest on airway management) after a targeted training in the Opendot Lab [14] under the supervision of Dr. Enrico Bassi (designer and manufacturer with a solid experience in healthcare).

We printed the single components of the tTNI with the print settings listed in Table 1 according to the technical features suggested by the manufacturer (please refer to their official website) [11].

**Table 1:** Setting of Prusa I3 MK3S for printing translaryngeal tracheostomy needle introducer parts with Verum T

Setting of Prusa I3 MK3S used	
Extruder temperature	265 °C
Plate temperature	115°
Fill density	50%
Fill texture	Gyroid
Number of top layers	5
Number of bottom layers	5
Brim (only for the arm)	> 10 mm

During the printing of the individual parts of the device, some minor problems were encountered which required specific technical solutions. First, the new filament tended to bend and break away from the printing bed, especially when long and thin parts were printed, such as the tTNI arm. We solved this problem based on three technical modifications: a) increased the printing bed temperature to 115 °C, b) used a specific spray glue for 3D (Dimafix, Dima 3D, Valladolid, Spain) [15], and c) used a brim for the arm of at least 1 cm.

**Fig. 6:**
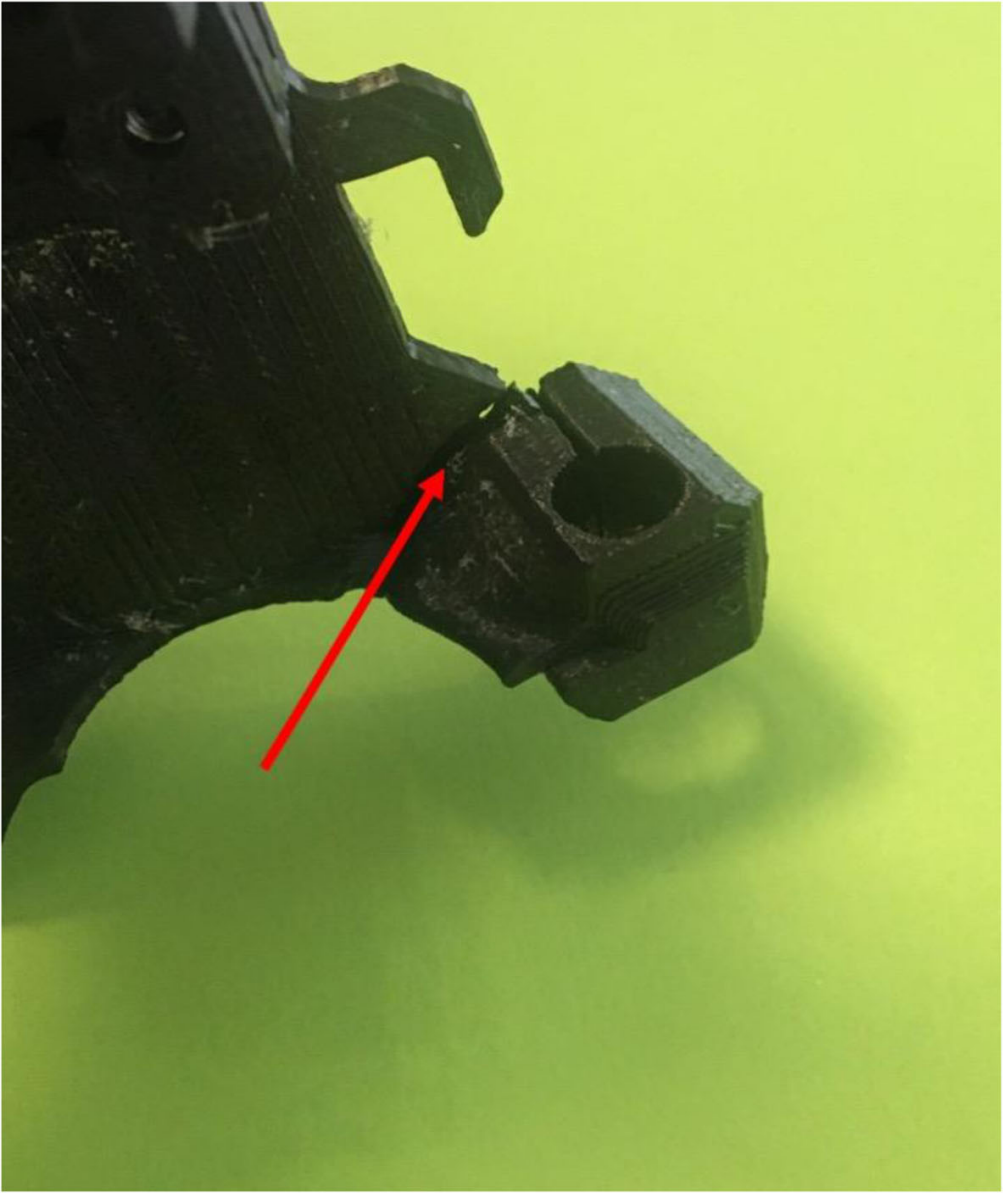
Photograph of the melted PINDA sensor

The second problem we encountered during printing with Verum T was the melting of a part of the extruder owing to the extreme temperatures (265 °C) necessary to melt the filament. To print the Verum T, we upgraded the Prusa I3 MK3 device to an MK3S after melting of the support of the PINDA (Prusa INDuction Autolevelling) sensor (Prusa Research, Fig. 6).

It is possible to avoid the repetition of this problem by limiting the 24 h use of the printer and by programming pauses of approximately an hour between the printing of one piece and the next one.

## Discussion

The reasons for which we decided to improve the translaryngeal tracheostomy (TLT) and preferred to choose additive manufacturing techniques to create all the versions of the new device included the following. First of all, in our opinion, the TLT was the first choice of procedure owing to its intrinsic safety (single dilatation of the neck from the inner to the outer parts of the trachea), especially for patients who faced an increased bleeding risk (e.g. patients who underwent anti-coagulation therapy for intra-aortic balloon pumps, or extracorporeal membrane oxygenation, or anti-platelet therapy, or those with thrombocytopenia).

The choice of turn on additive manufacturing techniques, mainly 3D printing, allows physicians to directly manage the prototyping process of a new medical device, making this process completely independent. In addition, the speed of the prototyping process and testing of each piece allows faster creation of a prototype compared with traditional industrial methods.

In daily practice, specialists often identify technical needs that can be solved by creating a new device. In the past, the procedure used to realise a prototype of a new device consisted of two main steps: conception of the device and production of the first prototype, which was usually entrusted to specialised companies. Currently, 3D printing offers an important opportunity to accelerate production of a new device by moving the production site of the prototype from specialised laboratories to the workplace of a single specialist. It has been emphasised that prototyping of new devices is expected to be the main application field of 3D printing in medicine [16, 17]. In our experience, 3D printing has been used for all the prototyping steps of the new tTNI device.

Finally, the new biomedical filaments offer physicians with endless creation and modelling possibilities. Furthermore, the relatively low cost of these remarkable 3D printers and biomedical filaments offer an undoubted advantage.

### Study limitations

As stated, the main limitation of this study is the lack of data pertaining to actual use on patients, pending the authorization of use in clinical trials by our Ethics Committee. Mannequin tests are insufficient to guarantee the accuracy and safety of the device made with the new thread. The absence of mechanical tests on the devices before and after the sterilization procedures may be a second limitation.

Outside of this specific study, in our opinion, there are two general main limitations regarding the use of additive manufacturing techniques. The need to train physicians in computer-aided design drawing (it is very difficult to find technical drawing courses for physicians) and in the basic use of 3D printers (however, the solutions to main printing problems are generally easily available online).

Finally, to date, it is not clear which safety certification path should be adopted for new medical devices manufactured with 3D printing before their use in clinical settings.

### Future perspectives

At the moment, it is not possible to proceed with clinical trials for two major reasons. First, ethical approval is still pending as there are no clear rules and laws on the safety certifications of 3D printed devices.

Second, it is particularly difficult to find the standard TLT kit used in Fantoni’s method because the manufacturer (Covidien-DAR; Medtronic, Minneapolis, MN, USA) has stopped its production.

Once we receive approval from our Ethics Committee, we will proceed with the experimentation of the tTNI on patients using the last few TLT kits left in our stock.

We strongly believe in the safety features of Fantoni‘s TLT technique [8, 9], which has been performed for several years without complications. Additionally, in view of the potential of additive manufacturing, we have started to work with the staff of Opendot Lab to verify the feasibility of realising a new TLT kit (version 2.0) made entirely with additive manufacturing technologies.

## Supplementary Information

**Additional file 1.** Supplemental_Italian patent.

**Additional file 2.** Supplemental_Verum_T_Cert.

**Additional file 4.** Supplemental_exagon.

**Additional file 5.** Supplemental_TNI arm.

**Additional file 6.** Supplemental_snap.

**Additional file 7.** Supplemental_head.

## Data Availability

Not applicable.

## Abbreviations

3D: Three-dimensional
PLA: Polylactic Acid
TLT: Translaryngeal Tracheostomy
tTNI: translaryngeal Tracheostomy Needle Introducer
