# Supplementary material for: The key role of 3D printing and the new medical sterilizable threads in the development of the translaryngeal Tracheostomy Needle Introducer
Source: 3D Print Med. 2021 May 12;7:14. doi: 10.1186/s41205-021-00104-w (PMC8117544; doi:10.1186/s41205-021-00104-w)
Supplement: Supplementary file 2 — Additional file 1. Supplemental_Italian patent. [file 41205_2021_104_MOESM1_ESM.pdf]

Loredana Guglielmetti Firmato da: uibm-brevetti
Roma. 16 luglio 2019

mise.AOO_PIT.REGISTRO UFFICIALE.U.0184716.16-07-2019

Ministero dello Sviluppo Economico
Direzione generale per la lotta alla contraffazione
Ufficio Italiano Brevetti e Marchi

ATTESTATO DI BREVETTO PER INVENZIONE INDUSTRIALE

Il presente brevetto viene concesso per l'invenzione oggetto della domanda:

N. 102017000035827

TITOLARE/I:
- BASSI Enrico 20.0%
- FOTI Giuseppe 10.0%
- TERRANI Alessandro 70.0%

DOMICILIO:
Guella Paolo
Brevetti Dr.Ing. Digiovanni Schmiedt S.r.l.
via Aldovrandi 7
20129 Milano

INVENTORE/I:
- TERRANI Alessandro
- FOTI Giuseppe

TITOLO: Dispositivo per posizionare in modo ottimale un ago per tracheostomia rispetto ad un tubo endotracheale, ed apparecchiatura comprendente detto dispositivo connesso ad un tubo endotracheale

CLASSIFICA: A61M1604

DATA DEPOSITO: 31/03/2017

Roma, 16/07/2019

Il Dirigente della Divisione
Loredana Guglielmetti
