# Supplementary figures and images for: The key role of 3D printing and the new medical sterilizable threads in the development of the translaryngeal Tracheostomy Needle Introducer
Source: 3D Print Med. 2021 May 12;7:14. doi: 10.1186/s41205-021-00104-w (PMC8117544; doi:10.1186/s41205-021-00104-w)

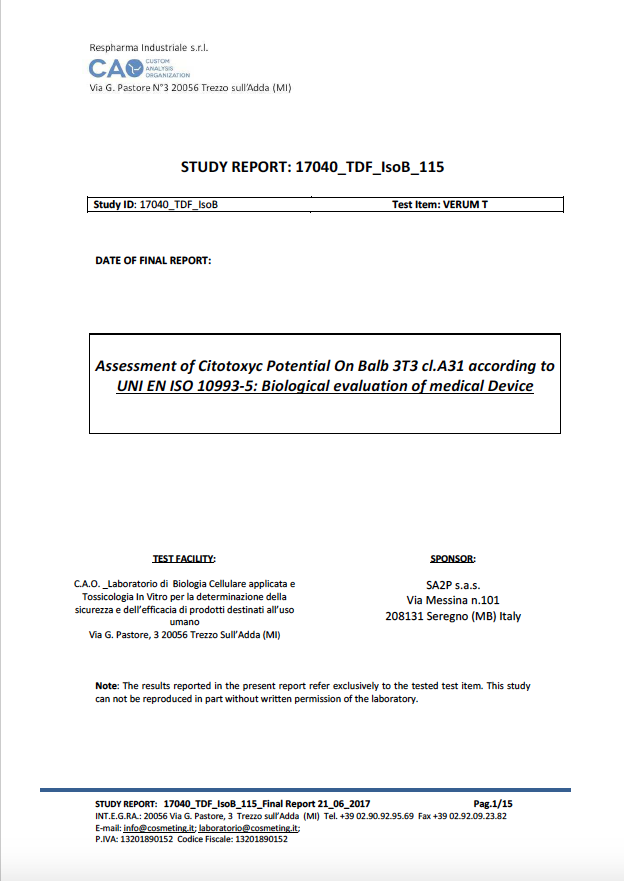

Supplement: Supplementary file 3 — Additional file 2. Supplemental_Verum_T_Cert. [file 41205_2021_104_MOESM2_ESM.png]

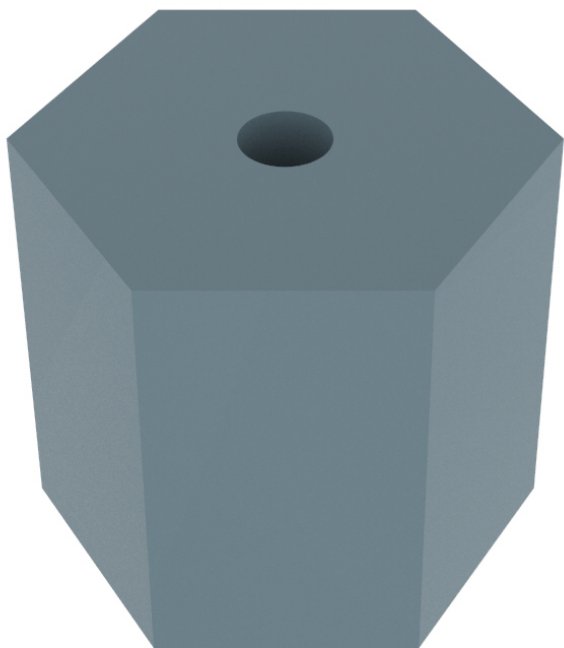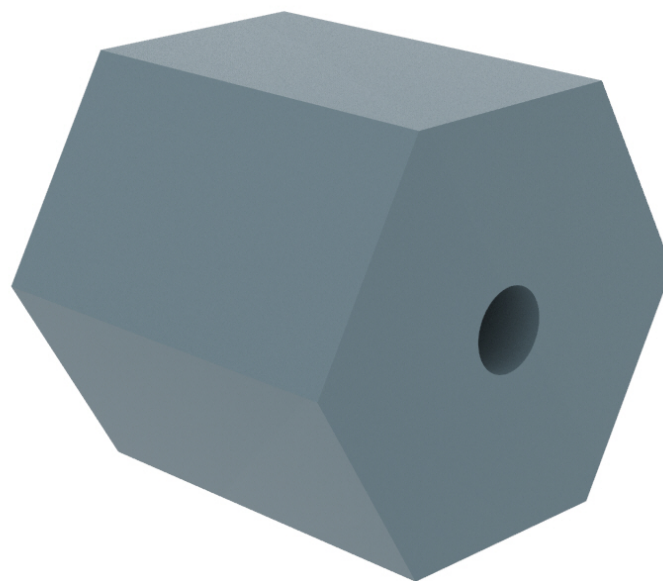

Supplement: Supplementary file 4 — Additional file 4. Supplemental_exagon. [file 41205_2021_104_MOESM4_ESM.pdf]

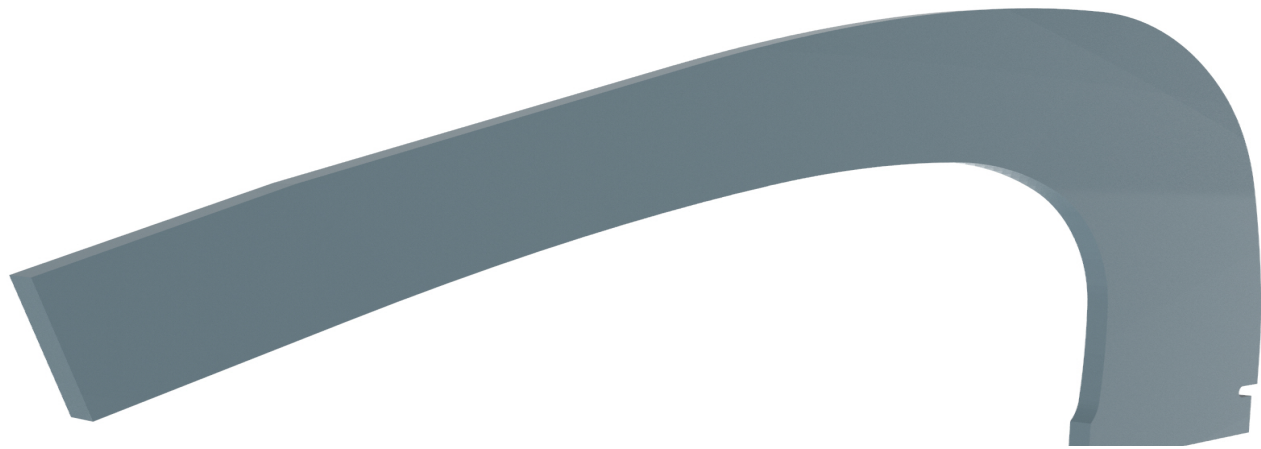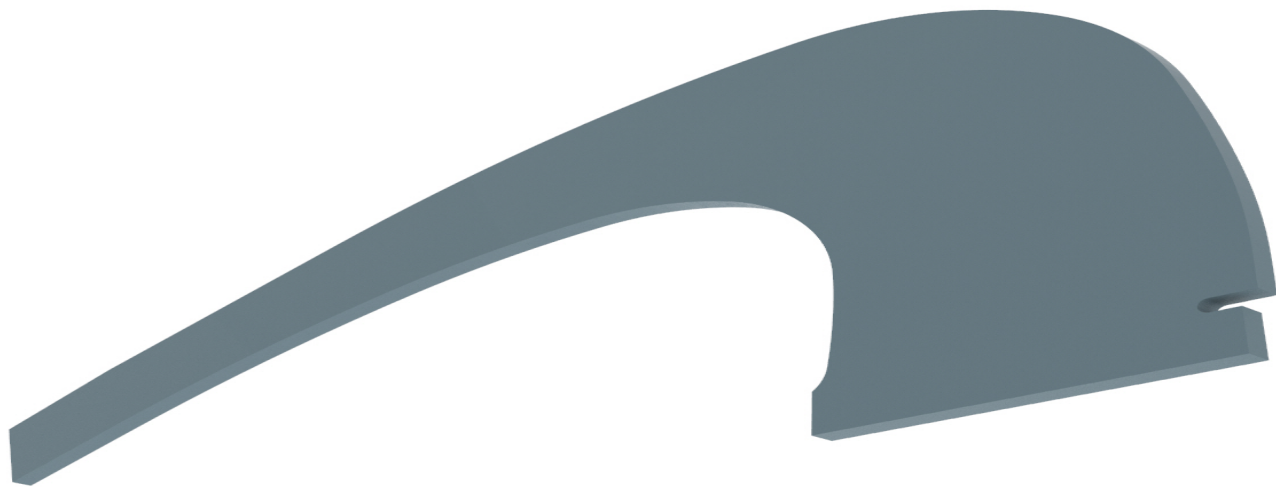

Supplement: Supplementary file 5 — Additional file 5. Supplemental_TNI arm. [file 41205_2021_104_MOESM5_ESM.pdf]

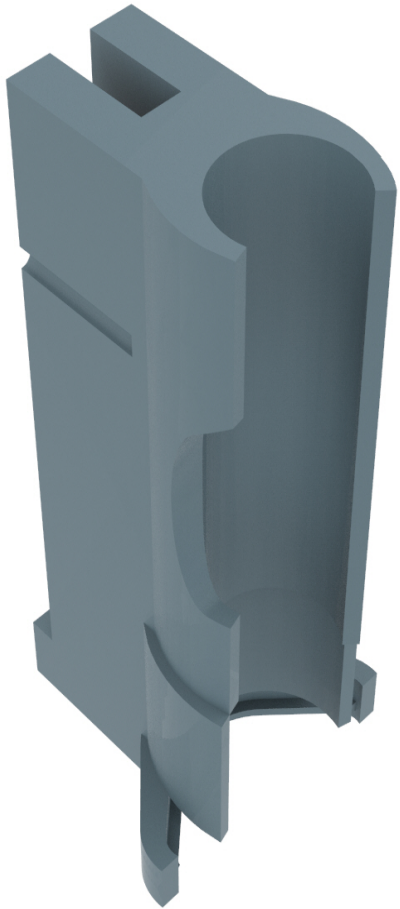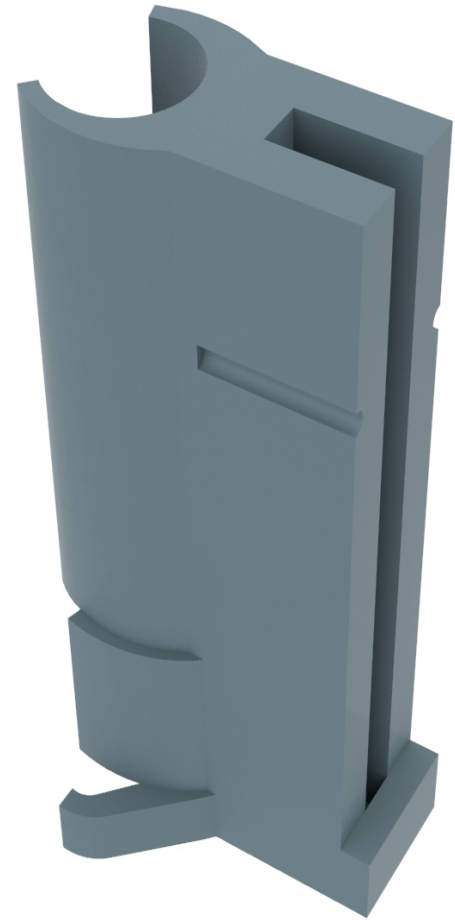

Supplement: Supplementary file 6 — Additional file 6. Supplemental_snap. [file 41205_2021_104_MOESM6_ESM.pdf]

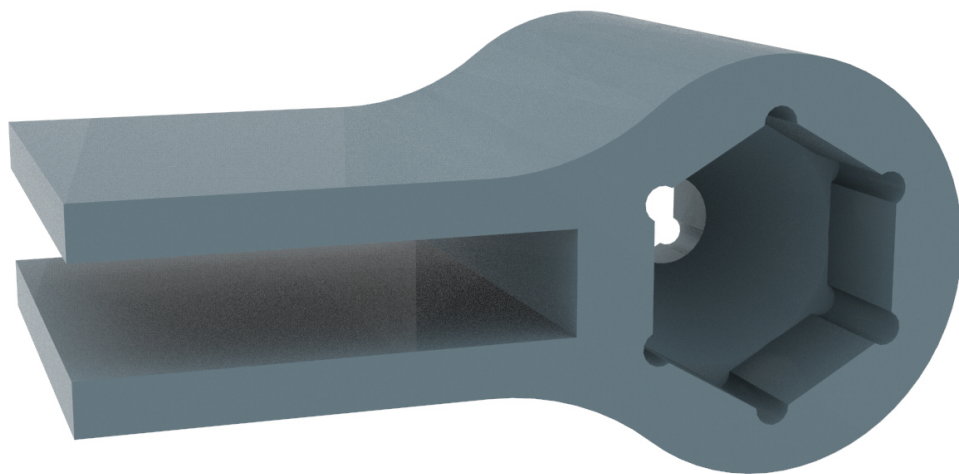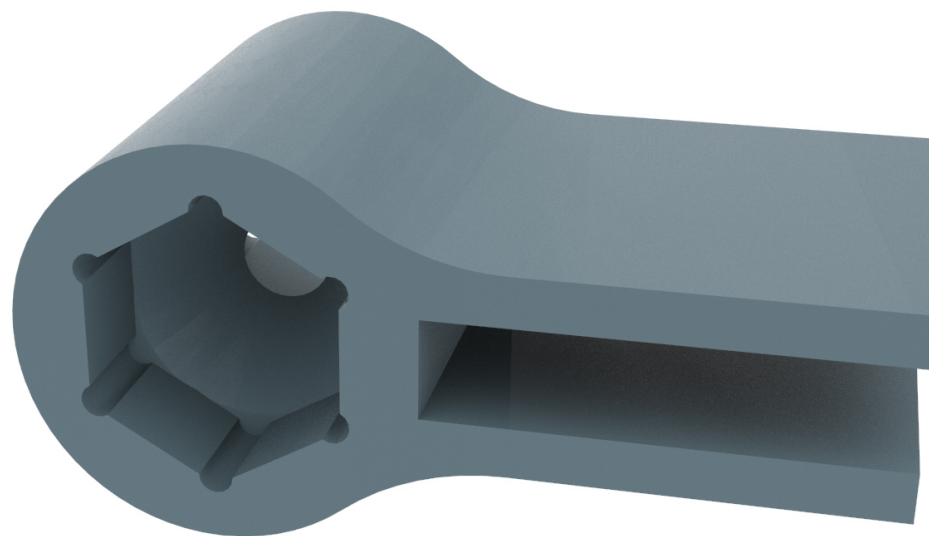

Supplement: Supplementary file 7 — Additional file 7. Supplemental_head. [file 41205_2021_104_MOESM7_ESM.pdf]
